# A Dedicated Multidisciplinary Growth and Feeding Clinic for Infants with Cleft Lip and/or Palate Demonstrates Need for Intervention

**DOI:** 10.1177/10556656241258687

**Published:** 2024-06-11

**Authors:** Kayla Prezelski, Daniel Villarreal Acha, Tuong-Vi Cindy Ngo, Caitlin Wilson, Vania Thrasher, Kandi Trevino, Cortney Van’t Slot, Rami R. Hallac, James R. Seaward, Alex A. Kane

**Affiliations:** 1 Department of Plastic Surgery, 12334 University of Texas Southwestern Medical Center, Dallas, TX, USA; 2 Analytical Imaging and Modeling Center, Children's Health, Dallas, TX, USA; 3 Department of Speech-Language Pathology, Children's Health, Dallas, TX, USA; 4 Department of Clinical Nutrition, Children's Health, Dallas, TX, USA

**Keywords:** cleft lip and palate, nutrition, feeding, intervention, outcomes

## Abstract

**Objective:**

A Growth and Feeding Clinic (GFC) focused on early intervention around feeding routines in patients with cleft lip and/or palate (CL/P) was implemented.

**Design:**

This study assessed the effect of preoperative feeding interventions provided by the GFC.

**Setting:**

Tertiary academic center.

**Methods:**

This study evaluated patients with CL/P who were cared for by the GFC and a control group of patients with CL/P. Weight-for-age (WFA) Z-score of less than −2.00 was used as a cutoff to classify patients who were underweight during the preoperative period.

**Main Outcome Measure:**

The number of underweight patients who were able to reach normal weight by the time of their cleft lip repair was used as the primary outcome measure.

**Results:**

Within both the GFC and control groups, 25% of patients with CL/P were underweight as determined by WFA Z-score. GFC patients who were underweight received more clinic visits (*P* < .001) and GFC interventions (*P* < .001) compared to GFC patients who were normal weight. At the time of cleft lip surgery, 64.1% of GFC underweight patients were normal weight compared to 31.8% of control group underweight patients (*P* = .0187).

**Conclusion:**

This study showed that multidisciplinary care provided by the GFC was able to target preoperative nutritional interventions to the highest-risk patients, resulting in double the percentage of patients who were of normal weight at the time of their cleft lip repair. These results provide objective proof supporting the assertion that multidisciplinary team care of the infant with cleft leads to measurable improvement in outcomes.

## Introduction

One of the greatest concerns for parents of infants born with cleft lip and/or palate (CL/P) is the establishment of a safe and efficient feeding routine.^1^ Feeding difficulties are prevalent in infants born with CL/P due to their inability to create the suction necessary to express adequate breast milk or formula from a nipple, with as many as 63% of infants born with CL/P experiencing feeding difficulties.^2^ Inadequate caloric intake due to feeding difficulties increases the risk of malnutrition, delays in the surgical timeline, and concerns with postoperative healing. In addition, patients with CL/P with feeding difficulties are at increased risk for poor growth and development, altered mother-child bonding, and aspiration leading to infections.^1,3,4^

Patients with CL/P represent a vulnerable population with unique nutritional needs. Previous research studies have investigated the prevalence of malnutrition in CL/P patients, and malnutrition as a risk factor in cleft lip and palate (CLP) surgery patients in both the United States and other countries.^5–7^ The incidence of failure to thrive (FTT) secondary to CL/P is found to range from 22.3% to 43% in the United States.^8–10^ Furthermore, Pandya et al. found that 38% of patients with CL/P had FTT at the time of surgery.^10^

Timely intervention to establish regular feeding routines and adequate caloric intake by a multi-disciplinary team is essential to support normal growth and development.^11^ There are currently no benchmarks or guidelines to direct clinicians in their decision-making regarding early identification of patients at the highest risk for chronic malnutrition or postoperative complications and the timing of initial cleft repair to maximize the chance of successful surgery.^8^

A specialized Growth and Feeding Clinic (GFC) focused on early intervention around functional feeding routines in patients with CL/P was implemented at our institution starting in 2016. The purpose of this study was to assess the effect of preoperative feeding interventions provided by our GFC in patients with CL/P compared to a control group of patients with CL/P from our institution. Evaluation of the interventions made by the GFC will allow insight into which strategies are most beneficial for preoperative nutritional support in this vulnerable population. Additionally, investigation of patient-specific factors such as CL/P phenotype, birth history, and demographic variables will help to determine patients who are at the highest risk for developing malnutrition.

## Methods

### Patients

This retrospective study included patients with CL/P who were seen at the Fogelson Plastic Surgery and Craniofacial Center Clinic and born between January 2011 to December 2022. A subset of patients, those born between January 2018 through December 2022, were seen in the multidisciplinary GFC. A control group of infants with CL/P born from January 2011 to December 2015 who were seen at our facility for cleft care prior to the implementation of the multidisciplinary care model of the GFC was gathered for comparison. The years 2016 and 2017 were excluded from the analysis due to the GFC being started and undergoing progressive changes during that time. Patients with microform cleft lip, submucous cleft palate (CP), and those with additional medical complexities i.e., with concurrent cardiac, neurologic, gastrointestinal, or other anomalies with potential confounding effects on feeding, were excluded from the study. Patients who were born at a gestational age of less than 35 weeks were also excluded due to the potential confounding effect of prematurity on weight gain. Patients who presented for an initial visit to the GFC after 100 days of life were excluded as this represents a critical time of intervention for the GFC. Patients with cleft surgeries performed at outside hospitals and those who underwent family relocation or change in providers during the perioperative period were also excluded.

This protocol was reviewed and approved by the institutional review board at our institution (STU-2023-0692).

### Growth and Feeding Clinic Typical Assessment

The Fogelson Plastic Surgery and Craniofacial Center at Children's Health Dallas evaluates and treats around 65 new patients with CL/P per year. The subspecialty clinic named the GFC was initiated in 2016 and included a Speech-Language Pathologist (SLP), Physician Assistant (PA), and Nurse Clinic Coordinator. All patients undergo an initial diagnostic feeding consultation with an SLP within the first few weeks of life. The goal of each visit is to review special feeding systems (bottles/nipples) for patients with CL/P and position and pacing for feeding. At each GFC visit, the feeding specialists perform a feeding history, feeding assessment for sucking pattern with direct observation of feeding, and calculate the rate of weight gain. If there are weight gain concerns, the team consults a registered dietician who can further evaluate the nutritional adequacy of diet and growth trends, and make recommendations on breastmilk fortification, formula concentration, goal volumes, and formula concentration. Social work, Gastroenterology, and Otolaryngology consults are also made when appropriate.

### Clinic Visit Records

Records from all clinic visits were reviewed and recorded for each patient. Patient birth history, CL/P phenotype, and sociodemographic factors were documented. Date of visit, weight at the visit, and GFC-specific interventions including changes in bottle type, nipple flow rate, feeding position, formula calorie density, delay in surgery, and use of alternate means of nutrition were recorded. The number of specialty referrals to Nutrition and Dietetics, Gastroenterology, Otolaryngology (for airway concerns), Social Work, and for Swallow Studies made by the clinic and all nutritional status-related hospital admissions were documented. Referrals made to Orthodontics for nasoalveolar molding (NAM), Otolaryngology for myringotomy and tympanostomy tube placement, or Audiology were not included in the list of specialty referrals due to these department referrals being standard of care for all eligible patients presenting with CL/P at our institution.

### Determining Nutritional Status

World Health Organization (WHO) sex-specific weight-for-age (WFA) Z-scores of less than −2.00 standard deviations from the mean growth standard were used to classify patients who were underweight, i.e., at risk for malnutrition.^12^ WFA Z-scores less than −2.00 were used as a surrogate to represent malnutrition, as this diagnosis is based on reliable and valid clinical and anthropometric measures over time rather than a single weight measurement.^4,8^ This criterion for nutritional status was selected because Z-scores are a good tool for comparing nutritional status across age and sex, and previous studies have shown that this metric is sensitive to feeding difficulties resulting from insufficient caloric intake.^9,13^ In addition, WFA Z-scores are a more sensitive indicator of risk for morbidity than weight-for-length (WFL) Z-scores in infants under 6 months of age,^14^ which is the demographic of focus in this study.

**Figure 1.**
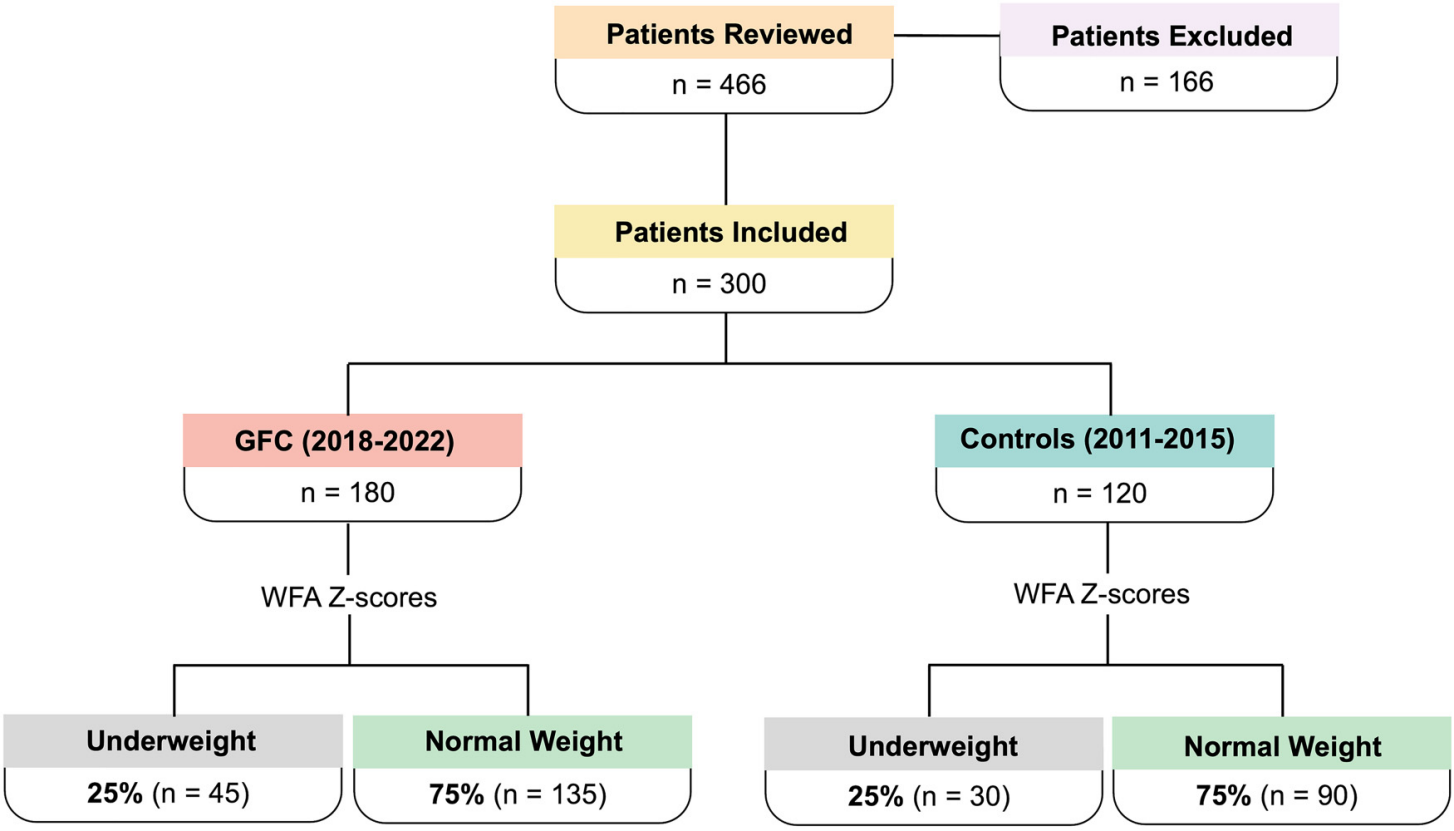
Study design flowchart.

WFA Z-score was calculated for all pre-operative clinic visit weights starting at day 14 of life in accordance with the American Academy of Pediatrics (AAP) expected return to birth weight.^11,15^ Patients with CL/P who fell below the WFA Z-score of −2.00 were classified as underweight, i.e., “at risk for malnutrition” vs. those who were of normal weight. For premature patients, corrected age was used for Z-score calculation with a baseline of 40 weeks gestation.^16^ (Figure 1).

### Primary Outcome Measure

WFA Z-score at the time of surgery was used to compare the number of underweight GFC patients who were able to reach normal weight at the time of surgery to the number of underweight control patients who were able to reach normal weight at the time of surgery.

### Statistical Analysis

Chi-square test was used to determine differences in the GFC group vs. control group demographic data. Group differences in continuous variables were analyzed using ANOVA with Tukey's post hoc pairwise comparisons and student's t-test where appropriate. A significance level of .05 was used for all tests.

## Results

### Patients

There were 300 patients with 1,488 unique pre-operative clinic visits with weight measurements after exclusion criteria. In the GFC group, 52.2% (n = 94) were males, and 47.8% (n = 86) were females. Patient diagnoses were 43.9% (n = 79) CLP, followed by 30.0% (n = 54) cleft lip (CL), and 21.1% (n = 47) isolated CP. In the control group, 50.0% (n = 60) were males, and 50.0% (n = 60) were females. Patient diagnoses were 45.8% (n = 55) CLP, followed by 29.2% (n = 35) cleft lip, and 25.0% (n = 30) isolated CP. The chi-square test showed that there were no statistically significant differences in any demographic variables between the GFC and control groups. Demographics are further detailed in Supplemental Table 1.

After nutritional status was determined at all preoperative visits, subgroups for underweight (WFZ Z-score < −2.00) vs. normal weight (WFA Z-score ≥ −2.00) patients were formed. The GFC group had 25% (n = 45) of patients who were underweight and 75% (n = 135) of patients who were of normal weight at preoperative visits. The control group had 25% (n = 30) of patients who were underweight and 75% (n = 90) of patients who were of normal weight at preoperative visits. There was no statistically significant difference between underweight vs normal weight patients in the two groups.

In the GFC group, underweight patients had an average birth weight of 2.9 ± 0.4 kg, and control group underweight patients had an average birth weight of 2.9 ± 0.7 kg. GFC normal weight patients had an average birth weight of 3.3 ± 0.5 kg, and control group normal weight patients had an average birth weight of 3.3 ± 0.4 kg. ANOVA showed a statistically significant difference in birth weight between the groups (*P* < .0001), with Tukey post-hoc analysis showing significant differences between the underweight and normal weight groups in both the GFC group (*P* < .0001) and the control group (*P* = .003). There was no significant difference in the age at first visit between any groups, with most patients being seen around the time or before they turned two weeks old. Weight at the patient's first visit was significantly different between the groups (*P* < .001), with Tukey post-hoc analysis showing differences between the underweight and normal weight groups in both the GFC group (*P* < .0001) and the control group (*P* = .0032). There was no difference in the age at cleft-lip repair (*P* = .3146) or cleft-palate repair (*P* = .4570). (Table 1)

**Table 1.** Weight and Age at Timepoints of Interest (Mean ± SD).

	GFC group		Control group		
	Underweight 25% (n = 45)	Normal 75% (n = 135)	Underweight 25% (n = 30)	Normal 75% (n = 90)	ANOVA *P*-value
Birth Weight (kg)	2.9 ± 0.4	3.3 ± 0.5	2.9 ± 0.7	3.3 ± 0.4	**<**.001*
Age at First Visit (days)	14.0 ± 12.0	20.1 ± 25.4	15.1 ± 11.2	13.3 ± 14.3	.0551
Weight at First Visit (kg)	2.8 ± 0.4	3.6 ± 0.8	2.9 ± 0.6	3.4 ± 0.6	**<**.001*
Age at CL surgery (days)	129.3 ± 34.6	121.3 ± 38.6	122.1 ± 30.0	134.9 ± 66.2	.3146
Age at CP surgery (days)	342.1 ± 63.4	341.7 ± 125.9	342.4 ± 68.5	325.0 ± 65.7	.4570

* and bold font indicates statistical significance.

Abbreviations: Standard deviation (SD); Cleft lip (CL); Cleft palate (CP).

### Growth and Feeding Clinic Visits and Interventions

**Figure 2.**
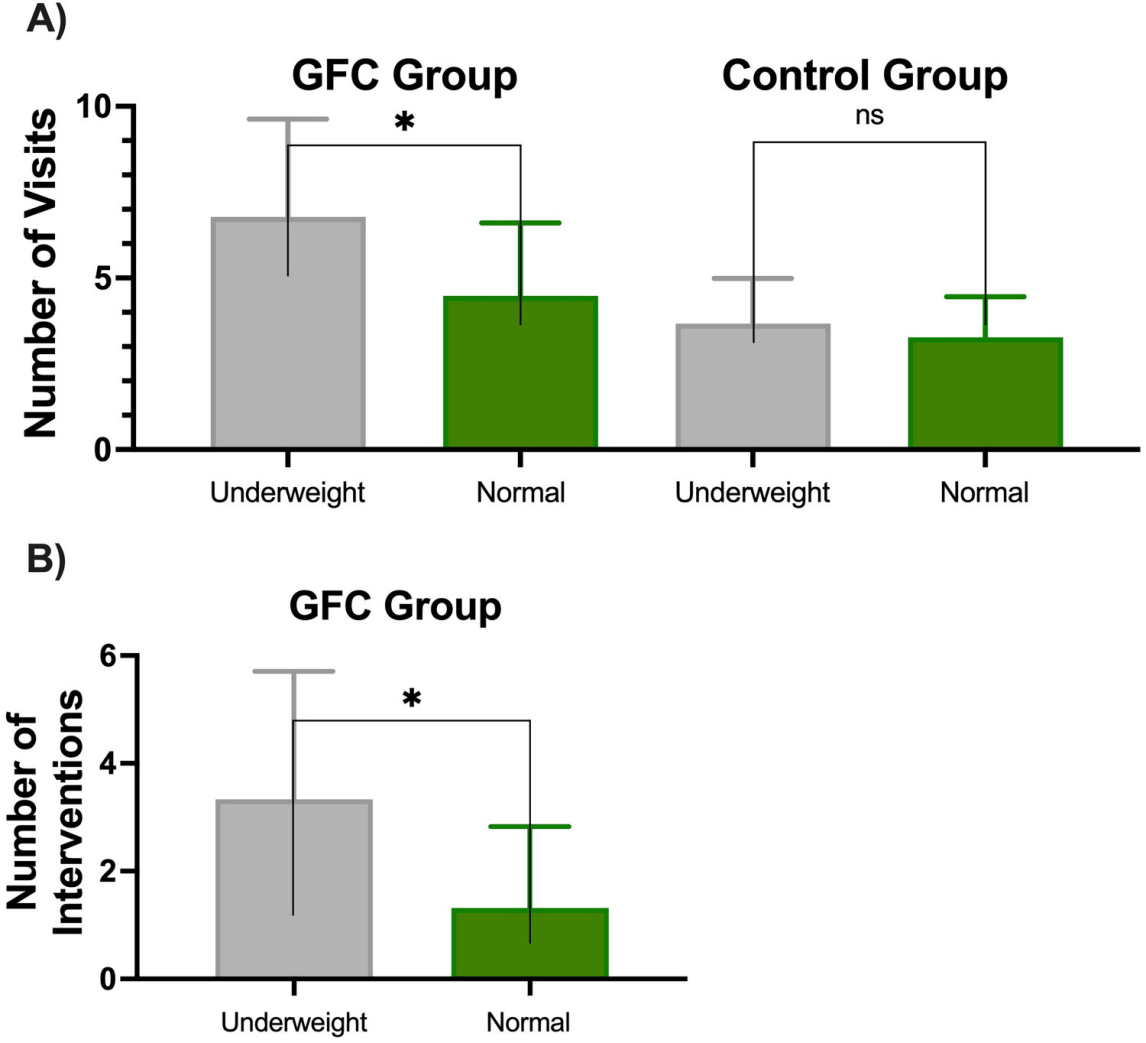
(A) Number of visits in underweight and normal weight patients in the GFC vs control groups. (B) Number of GFC interventions in underweight and normal weight patients in the GFC group. Error bars represent standard deviation; * represents a statistically significant difference.

GFC patients who were underweight had a higher average of 6.8 ± 2.9 clinic visits, compared to normal weight patients who had an average of 4.5 ± 2.1 clinic visits (*P* < .001) (Figure 2A). In the control group, underweight and normal weight patients had a similar number of visits, with underweight patients having 3.6 ± 1.3 visits, while normal weight patients had 3.3 ± 1.2 visits (*P* = .5564) (Figure 2A). GFC patients who were underweight received 3.3 ± 2.4 GFC interventions, compared to the GFC normal weight group who underwent 1.3 ± 1.5 interventions (*P* < .001) (Figure 2B). Interventions were not recorded in the control group due to these visits preceding the GFC start date and any formalized GFC visit and intervention process.

In the GFC group, interventions were performed for 97.8% of underweight patients, with the most common being increased formula calorie density and change in flow rate or feeding position. GFC normal weight patients were often offered similar interventions (64.4%). Eighty percent of GFC underweight patients received specialty referrals, with 68.9% of these patients receiving a referral to Nutrition and Dietetics for weight gain concerns. GFC patients of normal weight also benefitted from specialty referrals, with 22.2% of these patients receiving a Nutrition and Dietetics referral. (Table 2)

**Table 2.** GFC Interventions and Specialty Referrals. % (N).

	GFC Group	
	Underweight 25% (n = 45)	Normal 75% (n = 135)
**GFC Interventions**		
Patients with GFC Interventions	97.8 (44)	64.4 (87)
Change in feeding positioning	64.4 (29)	17.0 (23)
Increased formula calorie density	60.0 (27)	11.1 (15)
Bottle change	15.6 (7)	25.9 (35)
Flow rate change	64.4 (29)	38.5 (52)
Alternate means of nutrition	2.2 (2)	2.2 (3)
Modified surgical timeline	4.4 (2)	0.7 (1)
Other	2.7 (1)	6.7 (9)
**Specialty Referrals**		
Patients with Specialty Referrals	80 (36)	37.8 (51)
Nutrition and Dietetics	68.9 (31)	22.2 (30)
Social Work	26.7 (12)	12.6 (17)
Gastroenterology	4.4 (2)	5.2 (7)
Otolaryngology	11.1 (5)	10.3 (14)
SLP Swallow Study	6.7 (3)	5.2 (7)

Nutrition-related hospital admissions occurred in 8.8% of GFC underweight patients, and 6.6% of control underweight patients. This was higher than the 3.7% of GFC normal weight, and 4.4% of control normal weight patients, however, there was no statistically significant difference found between admission rates in the GFC (*P* = .163) or control (*P* = .629) group. The overall complication rate was increased in the GFC underweight group (22.2%) and the control underweight group (16.7%) compared to the GFC normal weight group (14.1%) and control normal weight group (12.2%). However, there was no statistically significant difference in complication rate within the GFC group (*P* = .198) or the control group (*P* = .629). Cleft phenotype differed significantly between the underweight and normal weight GFC patients (*P* = .002), however did not differ in the control group (*P* = .614). Insurance status did not differ significantly in the GFC (*P* = .131) or control (*P* = .296). (Table 3)

**Table 3.** Cleft Phenotype, Insurance status, Postoperative Complications, and Nutrition-related Hospital Admissions. % (N).

	GFC Group				Control Group			
	Underweight 25% (n = 45)	Normal 75% (n = 135)	χ^2^	*P*-value	Underweight 25% (n = 30)	Normal 75% (n = 90)	χ^2^	*P*-value
**Cleft phenotype**			12.65	.002*			0.979	.614
CLP	66.7 (30)	36.3 (49)			53.3 (16)	43.3 (39)		
CL	17.8 (8)	34.8 (47)			26.7 (8)	30.0 (27)		
CP	15.5 (7)	28.9 (39)			20.0 (6)	26.7 (24)		
**Insurance status**			5.638	.131			3.701	.296
Medicaid	73.3 (33)	54.1 (73)			50.0 (15)	67.8 (61)		
Private	24.4 (11)	39.3 (53)			46.7 (14)	28.9 (26)		
Self-pay	2.3 (1)	4.4 (6)			0.3 (1)	2.2 (2)		
Other	–	2.2 (3)			–	1.1 (1)		
**Complications**			1.658	.198			0.3846	.535
Unique Patients	22.2 (10)	14.1 (19)			16.7 (5)	12.2 (11)		
Fistula	70.0 (7)	52.6 (10)			60.0 (3)	72.7 (8)		
Dehiscence	40.0 (4)	42.1 (8)			40.0 (2)	27.3 (3)		
Infection	–	5.3 (1)			–	–		
Other	–	21.1 (4)			–	–		
**Nutrition-related hospital admissions**			1.944	.163			0.234	.629
	8.8 (4)	3.7 (5)			6.6 (2)	4.4 (4)		

* and bold indicates statistical significance.

**Figure 3.**
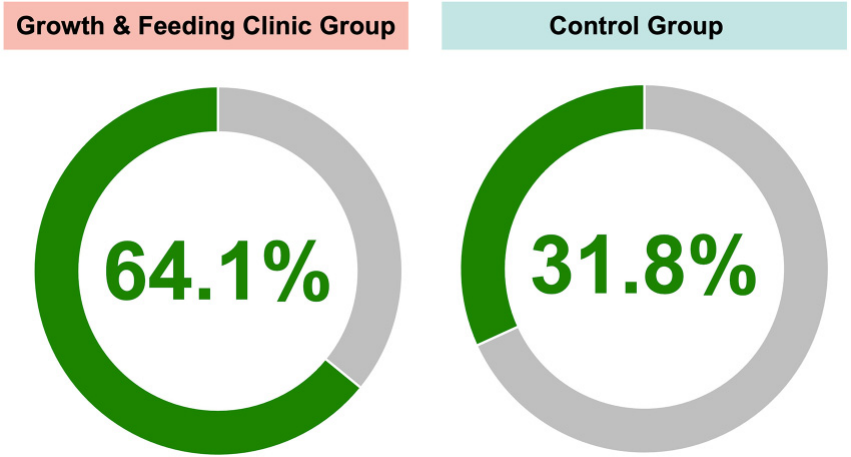
Weight outcomes at the time of surgery for underweight patients from the GFC and underweight groups.

In the GFC underweight group, there were 25 out of 39 patients (64.1%) who underwent cleft lip repair who were of normal weight by the time of their surgery (Figure 3). This was significantly higher than in the control underweight group, where only 7 out of 22 patients (31.8%) who underwent cleft lip repair were of normal weight by the time of their surgery (*P* = .0187). By the time of CP repair, 34 out of 37 patients (91.9%) in the GFC underweight group and 19 out of 22 patients (86.4%) in the control underweight group were of normal weight (*P* = .6614) (Figure 3).

## Discussion

Part of the stated mission of the American Cleft Palate-Craniofacial Association is to support multidisciplinary care.^17^ This can be a costly proposition compared to more diffuse types of care models, and consequently, insurance payors may not support such care, as they may not recognize the benefit. It can be difficult to precisely calculate the benefits of these costs, and this may lead to economic pressures on cleft teams. This study demonstrates the success of multidisciplinary care provided by a dedicated GFC which was able to target preoperative nutritional interventions to the highest-risk patients. Compared to a control group of patients with CL/P from our institution born before the implementation of this dedicated GFC, patients with access to care from the multidisciplinary GFC were two times more likely to reach normal weight by the time of their cleft lip repair (*P* = .0187).

Patients with CL/P are a vulnerable population, with this study indicating that 25% of patients in our CL/P cohort had WFA Z-scores consistent with a malnutrition diagnosis in the preoperative period, which compared to the WHO norms, is much greater than the expected 2.3% of patients from a normal population. A study by McKinney et al. focused on capturing the magnitude of malnutrition in patients with clefts found that WFA Z-scores of patients with CL/P were shifted downward relative to WHO norms from 0 to 6 months of life, with a peak incidence of 27.1% of patients with CL/P who were underweight occurring at 2-3 months,^9^ which is comparable to the current studies incidence of 25% underweight patients with CL/P in both the control and GFC groups.

Birth weight less than 3.0 kg was identified as a risk factor for patients with CL/P being underweight in both the GFC group (*P* < .001) and the control group (*P* = .003) (Table 1). Patients’ age at their first visit did not differ significantly between groups (*P* = .0551), however, their weight at the first visit was significantly lower in the GFC underweight (*P* < .0001) and control underweight (*P* = .0032) groups, emphasizing the importance of early intervention for these patients (Table 1).

Within the GFC patient group, those who were underweight had a higher number of clinic visits and GFC interventions compared to those who were of normal weight (*P* < .001) (Figure 2). This emphasizes the ability of the GFC to identify patients who are at high risk of becoming malnourished and to offer these high-risk patients more visits and interventions. Correctly identifying these high-risk patients is crucial to balance the number of visits and need for interventions with the increased burden of care while avoiding additional clinic visits in patients who would likely not benefit from additional visits and interventions.

Nearly all underweight GFC patients (97.8%) received interventions, while a high percentage of normal weight GFC patients (64.4%) also benefitted from interventions (Table 2). Eighty percent of underweight GFC patients and 37.8% of normal weight GFC patients were referred to Clinical Nutrition (Table 2). This data supports that patients with CL/P are a vulnerable population with well-documented feeding difficulties that are best addressed by specialists trained in feeding. Patients with CL/P who were of normal weight in the preoperative period required modifications to their feeding routines which may have included changing the feeding system, flow rate, feeding position, or pacing strategies. Additionally, patients with formula intolerance or parents with infant nutrition questions may have received a consult, regardless of their normal weight vs underweight status. Patients with a normal weight who had a poor weight gain velocity also received consults. These findings emphasize the benefit of having a multidisciplinary clinic available to all patients with CL/P at our institution, as we can proficiently intervene to help all patients feed safely and efficiently.

The cleft phenotype of CLP was identified as a statistically significant risk factor for being underweight in the GFC group (*P* = .002) (Table 3). Although not statistically significant, patients with Medicaid insurance status in the GFC group were more likely to be underweight in the preoperative period, which may reflect a broader socioeconomic influence on nutritional status that was not the focus of this study, however, has been detailed in other studies.^18^ Villavisanis et al. described that socioeconomic disparities in patients with CL/P may have a temporal component, with patients being at greatest risk in the early preoperative period between 4 and 8 weeks, coinciding with the period of interventions in this study's GFC.^18^

Previous studies by Escher et al. found that preoperative nutritional support with frequent feeding appointments was found to have a positive effect on acute malnutrition.^8^ The current study was able to expand upon this work by indicating specific interventions that were carried out by the GFC within this preoperative period. Escher et al. also emphasized the impact of preoperative nutritional status on postoperative complications. The current study did find an increased complication rate in patients who were underweight in the preoperative period compared to those who were normal weight, which along with Escher et al., supports the importance of the role of nutritional support in surgical wound healing. The current study does not differentiate between moderate and severe or acute and chronic malnutrition, however, the cumulative rates of patients with CL/P who were underweight at the time of initial cleft lip repair are comparable to our GFC group (∼40%) with both Escher et al. (39.8%) and Pandaya et al. (38%) who had persistent preoperative feeding and nutrition interventions leading up to surgery.^8,10^

Baylis et al. reported their quality improvement initiative aimed at improving feeding and growth in patients with CL/P that resulted in an institutional decrease in FTT hospital admission rate.^19^ Although the interventions in the study by Baylis et al. were centered around team communication, process tracking, and caregiver education, their study, and others^11,20,21^ support the idea that targeted interventions in this population can effectively improve preoperative nutritional status.

Limitations of this study include its retrospective nature as well as the single-institution design. A potential confounding factor for the number of visits in the normal weight and underweight patient groups is family psychosocial factors, which may have caused patients with a different risk profile to be seen for more visits than the typical patient course. In addition, neither missed appointments nor pediatrician visits were included in the number of clinic visits for any of the patient groups. Length-for-age Z-scores were not used in this study due to inconsistent and missing data. In addition, this study was focused on the preoperative period in a typical CL/P surgical repair schedule, i.e., the first 12 months of life, and because stunting develops over a period of many months, was beyond the scope of our investigation.^9^ Furthermore, advances in CL/P specific feeding, i.e., the Dr. Brown's Specialty Feeding System, could have contributed to the increase in normal weight by the time of surgery that was seen in our GFC group compared to the control group due to the time to market being around 2018. Notably, Madhoun et al. concluded that although patients with CL/P demonstrated improved feeding with the Dr. Brown's bottle, the patients were still behind their non-cleft counterparts in feeding.^22^ The fact that there was not an observed diminution in measured postoperative outcomes in this study might be the basis for criticism of the value proposition of our GFC. We believe that the benefit of halving the number of underweight patients at the time of surgery alone serves as proof of the benefit of the GFC concept. Nevertheless, one can speculate as to why no such difference occurred. This could reflect reporting differences in the pre-GFC (control group) vs. the GFC cohorts. It stands to reason that with the maturation of a cleft team, the reporting of complications becomes more stringent and accurate, although we cannot confirm this was the cause. To this point, the authors would like to acknowledge the limitation that some of the improvement in nutritional status seen in the GFC group may be attributable to external factors and confounding variables that were unable to be controlled for in a retrospective study design.

Observations gathered from this retrospective study on the implementation and outcomes of our institution's GFC have resulted in a formal change in our team practice patterns to more accurately identify patients at risk for malnutrition. Future work will include the addition of standardized length measurements at our clinic to assess for long-term outcomes regarding nutrition status. In addition, risk factors identified in this study including birth weight less than 3.0 kg, diagnosis of CLP, and Medicaid insurance status will serve as a framework to identify patients who should have a lower threshold for an increased number of clinic visits and GFC interventions. Furthermore, we will seek to further define the highest yield GFC interventions and better identify the patients who will benefit most from them based on rates of chronic vs. acute malnutrition, postoperative complications, and socioeconomic-based factors.

## Conclusion

In this study, we have demonstrated the necessity of this multidisciplinary care effort for patients with CL/P and unique nutritional needs. This study directly examined the effect of preoperative nutritional interventions in patients with CL/P on nutritional status at the time of surgery, postoperative complication rates, nutrition-related hospital admission status, and clinical interventions. In comparison to a control group of patients with CL/P born prior to the implementation of our institution's GFC, underweight patients cared for by the GFC were twice as likely to reach normal weight by the time of cleft lip repair (*P* = .0187).

## Supplemental Material

sj-docx-1-cpc-10.1177_10556656241258687 - Supplemental material for A Dedicated Multidisciplinary Growth and Feeding Clinic for Infants with Cleft Lip and/or Palate Demonstrates Need for Intervention Supplemental material, sj-docx-1-cpc-10.1177_10556656241258687 for A Dedicated Multidisciplinary Growth and Feeding Clinic for Infants with Cleft Lip and/or Palate Demonstrates Need for Intervention by Kayla Prezelski, Daniel Villarreal Acha, Tuong-Vi Cindy Ngo, Caitlin Wilson, Vania Thrasher, Kandi Trevino, Cortney Van’t Slot, Rami R. Hallac, James R. Seaward and Alex A. Kane in The Cleft Palate Craniofacial Journal
