# Supplementary material for: A Dedicated Multidisciplinary Growth and Feeding Clinic for Infants with Cleft Lip and/or Palate Demonstrates Need for Intervention
Source: Cleft Palate Craniofac J. 2024 Jun 11;62(8):1410–7. doi: 10.1177/10556656241258687 (PMC12198457; doi:10.1177/10556656241258687)
Supplement: sj-docx-1-cpc-10.1177_10556656241258687 - Supplemental material for A Dedicated Multidisciplinary Growth and Feeding Clinic for Infants with Cleft Lip and/or Palate Demonstrates Need for Intervention [file sj-docx-1-cpc-10.1177_10556656241258687.docx]

| **Supplementary Table 1**. Demographics % (N) | | | | | | |
| --- | --- | --- | --- | --- | --- | --- |
|  | **GFC Group** | | **Control Group** | | **Χ^2^** | **p-value** |
| **Sex** |  |  |  |  | 0.1271 | 0.7214 |
| Male | 52.2 (94) | | 50 (60) | |  |  |
| Female | 47.8 (86) | | 50 (60) | |  |  |
| **Cleft Phenotype** |  |  |  |  | 0.1224 | 0.9453 |
| CLP | 43.9 (79) | | 45.8 (55) | |  |  |
| CL | 30.0 (54) | | 29.2 (35) | |  |  |
| CP | 21.1 (47) | | 25.0 (30) | |  |  |
| **Race** |  |  |  |  | 5.811 | 0.1212 |
| Asian | 6.1 (11) | | 1.6 (2) | |  |  |
| Black | 10.6 (19) | | 11.7 (14) | |  |  |
| White | 74.4 (134) | | 71.7 (86) | |  |  |
| Other | 8.9 (16) | | 15.0 (18) | |  |  |
| **Ethnicity** |  |  |  |  | 0.0556 | 0.8137 |
| Hispanic | 50.5 (91) | | 49.2 (59) | |  |  |
| Non-Hispanic | 49.5 (89) | | 50.8 (61) | |  |  |
| **Insurance** |  |  |  |  | 1.129 | 0.7702 |
| Medicaid | 58.9 (106) | | 63.4 (76) | |  |  |
| Private | 35.5 (64) | | 33.3 (40) | |  |  |
| Self-pay | 3.9 (7) | | 2.5 (3) | |  |  |
| Other | 1.7 (3) | | 0.8 (1) | |  |  |

Abbreviations: Cleft lip and palate (CLP); Cleft lip (CL); Isolated cleft palate (CP)
